# Accurate Quantification of Lipid Species by Electrospray Ionization Mass Spectrometry — Meets a Key Challenge in Lipidomics

**DOI:** 10.3390/metabo1010021

**Published:** 2011-11-11

**Authors:** Kui Yang, Xianlin Han

**Affiliations:** 1 Division of Bioorganic Chemistry and Molecular Pharmacology, Department of Internal Medicine, Washington University School of Medicine, St. Louis, MO 63110, USA; E-Mail; kyang@wuchem.wustl.edu (K.Y.); 2 Sanford-Burnham Medical Research Institute, Orlando, FL 32827, USA

**Keywords:** electrospray ionization mass spectrometry, lipidomics, quantification, shotgun lipidomics

## Abstract

Electrospray ionization mass spectrometry (ESI-MS) has become one of the most popular and powerful technologies to identify and quantify individual lipid species in lipidomics. Meanwhile, quantitative analysis of lipid species by ESI-MS has also become a major obstacle to meet the challenges of lipidomics. Herein, we discuss the principles, advantages, and possible limitations of different mass spectrometry-based methodologies for lipid quantification, as well as a few practical issues important for accurate quantification of individual lipid species. Accordingly, accurate quantification of individual lipid species, one of the key challenges in lipidomics, can be practically met.

## 1. Introduction

A cellular lipidome is a very complicated system, potentially comprised of hundreds of thousands of individual lipid molecular species [1,2]. These species are classified into different classes based on their polar head groups [3] and subclasses of a class according to the linkages of the aliphatic chains [4,5]. Different classes, subclasses, and molecular species of lipids play a multitude of diverse roles in cellular functions ranging from membrane structural components to lipid second messengers [6]. Any perturbation of a biological system is expected to give rise to changes in the abundance and/or composition of the lipid pool. The newly-emerged discipline, lipidomics, is to determine these changes, to locate the place(s) (subcellular membrane compartments and domains) where the changes occur, to delineate the biochemical mechanisms underpinning the changes, to determine the relationship of the changed lipids with other neighboring lipids or proteins in a spatial and temporal manner, *etc.* [7]. In the field of lipidomics, accurate quantification of individual lipid species is a major, yet challenging component.

Quantification in omics generally falls into two categories, *i.e.*, relative and absolute quantifications. The former measures the pattern change of the lipid species in a lipidome, which can be used as a tool for readout after stimulation or for biomarker discovery. The latter determines the mass levels of individual lipid species, and then each individual lipid subclass and class of a lipidome. Measurement of the changed mass levels of individual lipid class, subclass, and molecular species is critical for elucidation of biochemical mechanism(s) responsible for the changes and for pathway/network analysis in addition to serving as a tool for readout after stimulation or for biomarker discovery. Thus, only the latter case is extensively discussed.

It should be pointed out that the word “quantification” to chemists and biochemists might lead to different expectations. To a chemist, quantification must be very “accurate”. All attempts in each step of a quantitative analysis from sampling to data processing would be made to achieve the highest degree of accuracy and/or precision possible. Therefore, error propagation can be pre-estimated and controlled. To a biochemist, the accuracy expectation for quantification is relatively loose since many uncertainties in the analysis of biological samples are inevitably present in the whole process from sampling, sample preparation, and analysis. For example, the variations present in sampling of biological samples could be substantial and surpass any analytical errors. Therefore, employing some kinds of compromise methods or correction factors for quantification of a particular category of compounds might be acceptable and practical. Moreover, a statistical analysis of the data obtained is usually essential for quantification or comparison. Unfortunately, different statistical methods could lead an analyst to having different conclusions, particularly if the accuracy and/or reproducibility for acquiring analytical data are also relatively low. Therefore, while the accuracy of quantification is relatively loose, the higher accuracy and better reproducibility that a platform for quantification of lipid species can achieve, the more meaningful results can be obtained and eventually the more resources and efforts can be saved.

Many modern technologies (including mass spectrometry (MS), nuclear magnetic resonance spectroscopy, fluorescence spectroscopy, chromatography, and microfluidic devices) have been used in lipidomics for quantification of lipid species in biological systems [8]. Clearly, electrospray ionization mass spectrometry (ESI-MS) has evolved to be one of the most popular, powerful technologies for quantitative analyses of individual lipid species [9-12]. There are two major platforms commonly employed for quantitative lipid analysis through ESI-MS, *i.e.*, methods based on LC-MS and direct infusion. Herein, the principles, advantages and possible limitations of each methodology, as well as a few practical issues for accurate quantification of individual lipid species are discussed.

## 2. Principle of Quantification of Lipid Molecular Species with Mass Spectrometry

Quantification of the concentration of an analyte with MS analysis, in principle, employs a correlation between the concentration and the ion intensity of the analyte which is linear within a pre-determined linear dynamic range:

(1) $$\text{I}={\text{I}}^{\text{app}}-b=a*c$$

where c is the concentration of the analyte; I^app^ is the apparent ion intensity of the analyte measured with MS; *b* is the spectral baseline resulting from baseline drift and/or chemical noise and can be determined as described recently [13]; I is the baseline-corrected ion intensity of the analyte (*i.e.*, the actual ion intensity); and *a* is the response factor. When I^app^ ≫ *b* (e.g., S/N > 10), I ≈ I^app^; otherwise, spectral baseline correction is required to obtain the actual ion intensity I from the measured apparent ion intensity I^app^ of the analyte. If a constant response factor *a* could be determined for the analyte, quantification of its concentration (within linear dynamic range) would be simply obtained from its baseline-corrected ion intensity with Equation 1. However, the ion intensity of an analyte measured with MS could be easily affected with even minor alterations in the conditions of analyte ionization and instrumentation and therefore might be varied or irreproducible for an identical analyte at a fixed concentration. Moreover, most of the alterations could not be controlled or might not even be noticed. Accordingly, it would be difficult to determine the constant response factor for an analyte of interest, thus direct quantification from Equation 1 would be mostly impossible.

Therefore, quantification of an analyte with MS analysis usually requires comparisons to either an external or internal standard that has a similar structure to the analyte (e.g., its stable isotopologue). When an external standard is used, a calibration curve is established with the external standards at a series of concentrations each of which should be analyzed under identical conditions that will be applied to the MS analysis of the analyte of interest. When an internal standard is used, the standard is added at the earliest step possible during sample preparation, and is analyzed simultaneously with the analyte.

The advantage of using an external standard is that there is no concern of the potential overlapping of extraneously added standards with endogenous molecular species. However, control of the analyses of external standard and analyte of interest under identical conditions is generally difficult. For example, the multiple steps involved in sample preparation (including separation) may lead to differential recovery and carryover from sample to sample; the varied composition of the analyzed solution due to the use of gradients or the presence of co-eluents during chromatographic separation may contribute to differential ionization conditions from run to run; and the varied spray stability during ESI-MS analysis and other factors may lead to differential ionization efficiency from time to time. Therefore, use of external standards alone is normally not the best choice for the analysis of a complex system particularly associated with a complicated process such as the global analyses of the cellular lipidome. The advantage of using an internal standard is its simplicity and accuracy resulting from its being processed and analyzed simultaneously with the analyte of interest. However, selection of an appropriate internal standard might be difficult because different systems may need different standards and specifically synthesized standards may be necessary to avoid any potential overlap with endogenous species in the analyzed system.

When a standard is used for quantification of the concentration of an analyte, it is derived from Equation 1 that:

(2) $${\text{I}}_{\text{u}}/{\text{I}}_{\text{std}}=(a_{\text{u}}/a_{\text{std}})*({\text{c}}_{\text{u}}/{\text{c}}_{\text{std}})$$

where I_u_ and I_std_ are the actual or baseline-corrected ion intensities of the analyte and the selected standard, respectively; c_u_ and c_std_ are the unknown concentration of the analyte and the known concentration of the standard, respectively; and *a*_u_ and *a*_std_ are the response factors of the analyte and the standard, respectively, under the experimental conditions. If the analyte and the selected standard have identical response factors (*i.e.*, *a*_u_ = *a*_std_), then the concentration of the analyte is determined from the following simplified equation with no need of determining the response factors.

(3) $${\text{c}}_{\text{u}}=({\text{I}}_{\text{u}}/{\text{I}}_{\text{std}})*{\text{c}}_{\text{std}}$$

Selection of the stable isotopologue of the analyte as the internal standard for its quantification would perfectly satisfy the requirement of having identical response factors because the stable isotopologue has the same structure and property as the analyte (e.g., the same recovery and same ionization efficiency) and the internal standard is processed and analyzed at the same time as the analyte. However, this approach is impractical if not impossible to analyze numerous species of interest in a complex system such as a cellular lipidome [14]. In the field of lipidomics, it was proved that individual lipid species in a polar lipid class could possess nearly identical response factors in the low concentration region due to two facts [15-17]. One is that the ionization efficiency of different lipid species in a polar lipid class is predominantly dependent on their identical charged head group while their differential acyl chains including the length and unsaturation only minimally affect the ionization under certain conditions. The other is that lipids at high concentration tend to form aggregates that are poorly ionizable. The formation of lipid aggregates is acyl chain-dependent, which in turn leads to differential response factors for individual lipid species with varied acyl chains (e.g., differential chain length and unsaturation) [16]. Accordingly, polarity and low concentration requirement are critical to achieve linear response by ESI-MS for accurate quantitation of lipid species with comparison to an internal standard. Since identical response factors are not valid for individual lipid species in non-polar lipid classes (e.g., triacylglycerol) even in the low concentration region, the response factors for individual non-polar species or a correlation between response factors and acyl chain length and unsaturation needs to be pre-determined for accurate quantification [18]. Alternatively, these non-polar lipid classes have to covert to polar lipids through derivatization prior to their quantification.

## 3. Quantification of Lipids with LC-Coupled ESI Mass Spectrometry

The use of the combination of MS with chromatographic separation for quantitative analysis of lipids needs to meet at least one of the following requirements to ensure the accuracy of quantification. First, a standard curve of a particular lipid species of interest is established under identical experimental conditions to the sample analysis. Second, a stable isotope-labeled internal standard of a lipid species of interest is available. Third, it is validated that ionization efficiency of each individual species of a polar lipid class is identical under experimental conditions after considering the appropriate correction factors, and the fragmentation kinetics of each species is identical if tandem mass spectrometry (e.g., selected reaction monitoring (SRM) or multiple reaction monitoring (MRM)) is employed. Among the methods based on the three requirements, the first method or its variants has been used broadly in practice [19,20]. The second one is impractical for quantification of numerous species in a lipidomic approach while studies with one or limited species have been widely reported [21]. The third one makes it possible to use one standard (or one standard curve) to quantify individual lipid species in a class but is mostly used for a rough quantitation with less accuracy compared to the former two methods [22-24].

To perform quantitative analysis of lipids by LC-MS, the limit of detection, the standard curves and their linear dynamic ranges are generally pre-determined before sample analysis. In practice, at least one internal standard for each lipid class is generally included in the sample to normalize the differential ionization efficiencies from different lipid classes that possess differential head groups [25,26]. Accordingly, each of the ion peaks of individual species is first normalized to the internally added control species from the same class prior to comparison with the appropriate standard curve(s) for quantification. This approach reduces the variability of quantification by diminishing the effects of the variations of chromatographic separation conditions and/or ESI-MS conditions that can dramatically alter the detected absolute ion counts of a particular species but much less affect the relative ion counts of the species obtained by normalizing to the ion counts of the internal standard detected under identical conditions if co-eluted or nearly identical conditions if eluted at different times.

Two major LC-MS techniques for quantitative analysis of lipids include selected ion extraction (SIE) and SRM. The SIE approach utilizes a survey scan for quantification while the SRM (or MRM) approach performs tandem MS and monitors a particular pair (or pairs) of precursor/product ions at a specified elution time for quantification. The SIE approach is usually used for “global” lipid analysis where mass spectra are acquired continuously during a chromatographic separation. The particular ions of interest are extracted from the acquired data array and the reconstituted peak of each extracted ion can be quantified with comparisons to either the reconstituted ion peak of an internal standard or a standard curve of the particular ion established under identical experimental conditions. The advantage of this approach is its simple instrumentation because no tandem MS is required but the specificity of the extracted ion to the targeted species is always a concern. A high mass accuracy/resolution MS would be preferable in this approach. In contrast to SIE, SRM is generally more specific than the SIE approach if the monitored precursor-product transition is specific to the targeted precursor eluted at a specified elution time while co-eluents have no interfering transitions. However, this approach requires previous knowledge of the transition from a targeted precursor ion to its specific fragment ion and the numbers of transitions that can be monitored during column elution (“on the fly”) are limited. An instrument possessing a high duty cycle capability is therefore crucial to employ this approach for quantification of multiple species. In comparison to SIE (*i.e.*, LC-MS) approach, SRM (*i.e.*, LC-MS/MS) approach has not only higher specificity but also higher sensitivity [20]. The former is due to the specific monitoring of a pair of transitions while the latter is due to the marked noise reduction through filtering with tandem MS.

These LC-MS techniques are theoretically suitable for many stationary phases (normal-phase, reversed-phase, ion exchange, hydrophilic interaction, *etc.*) as long as the elution conditions are effectively coupled with the mass spectrometer. In practice, LC-MS has been employed for many applications in lipid identification and quantification. For example, Hermansson and colleagues separated over 100 lipid species employing a diol-modified silica column and identified and quantified these species through two-dimensional maps of elution time and masses of the ions [27]. Sommer, Byrdwell, and others have employed dual LC coupled with MS (e.g., fractionation of lipid classes by normal-phase LC-MS followed by reversed-phase LC-MS or LC-MS/MS) to analyze lipid species in different classes [28,29]. Masukawa and colleagues have employed normal-phase LC-MS with a non-linear gradient to quantify over 182 ceramide species in human stratum corneum [30]. Merrill and colleagues have employed normal-phase and reversed-phase LC-MS to identify and quantify lipid species in sphingolipidomes [5]. Many researchers have broadly employed reversed-phase LC in conjunction with negative ion ESI-MS/MS to identify and quantify eicosanoids from biological samples [21,31]. Recently, Bohlinger, *etc.* have developed a charge-switch methodology employing derivatization to markedly increase the sensitivity of eicosanoid analysis by coupling HPLC with positive-ion ESI-MS/MS [32]. Many researchers have employed ultra-performance LC (UPLC) to replace the sequential separation with normal- and reversed-phase HPLC and succeeded in analysis of different lipid classes including phospholipids, sphingolipids, and triacylglycerols [23,33-35].

It should be recognized that discovery and quantification of low and very low abundance lipid species is one of the major advantages of the LC-MS compared to direct infusion-based MS. This is because chromatographic separation can reduce interferences from the high abundance lipid species and simultaneously enrich the low abundance species to allow their identification and accurate quantitation by MS.

It should also be emphasized that although chromatographic separation can enrich low-abundance lipid species and eliminate the inferences from the high abundance species during ionization, LC-MS has inherent difficulties. First, although the chromatography partially obviates the effects of “ion suppression” by eliminating lipid-lipid interactions between different lipid species (*i.e.*, the hetero-interaction) via column separation, there is a large (up to 1000-fold) increase in the lipid-lipid interactions between same lipid species (*i.e.*, the homo-interaction) due to the column enrichment or concentration that can affect the linear dynamic range of quantitation. If there are large concentrations of ions present in mobile phase (e.g., for ion-pairing or enhanced separation), additional ion suppression is generated. Moreover, when normal-phase LC is employed to separate lipid classes, discrete lipid species in a class are not uniformly distributed in the eluted peak due to their differential interactions with the stationary phase. When reversed-phase LC is employed to resolve individual lipid species in a class, the relatively polar mobile phase at the initial stage of the gradient can induce solubility problems in a species-dependent manner leading to differential apparent ionization efficiency while the applied gradient can also introduce alterations in ionization efficiency and cause ionization instability during elution. Furthermore, there are concerns over differential loss of lipid species and carry-over effects on the column [36]. Finally, the use of multiple steps in sample preparation, chromatographic separation and MS analysis can introduce experimental errors in each step that are propagated during processing. These errors are unlikely fully correctable by the standard curves that are generally established separately and unlikely under “identical” conditions to sample analysis. These limitations and other practical difficulties limit the utilization of LC-MS for high-throughput, large scale quantitative analysis of lipids; however, as exemplified above and by many reviews, there are many applications of LC-MS in disease-based discovery, and identification and quantification of novel lipids, particularly those present in extremely low abundance in a small scale [10,17].

## 4. Quantification of Lipids with Direct Infusion-Based ESI Mass Spectrometry

There is a misconception consistently stated in the literature that ion suppression present in the analysis of complex lipid mixtures precludes quantification by any method that uses direct infusion. This concept is misleading because it only holds true when inappropriate conditions for MS analysis are employed (e.g., high concentrations when the formation of lipid aggregates precludes meaningful quantification). If one uses concentrations outside of the linear dynamic range of a mass spectrometer, neither approach (LC-MS based, or direct infusion-based, or others) can work properly.

In contrast to the LC-MS based approaches, the direct infusion-based MS analysis first allows a full mass spectrum that displays molecular ions of individual species of a class in the infused solution. Next, many tandem mass spectra can be acquired for detailed structural and quantitative analysis under a constant concentration of solution during direct infusion and without the time constraints encountered with LC-MS during its “on the fly” analysis. These tandem MS approaches applied include precursor ion scanning (PIS) of particular fragment ions, neutral loss scanning (NLS) of specific neutral loss fragments, and product ion scanning of molecular ions of interest, each of which has been widely applied in direct infusion-based MS to facilitate the high-throughput analysis of a cellular lipidome on a global scale. The direct infusion-based MS analysis of lipids has been termed shotgun lipidomics. There are at least three platforms for shotgun lipidomics: (1) lipid class diagnostic MS/MS-based technologies; (2) high mass accuracy/high mass resolution MS-based technologies; and (3) multi-dimensional MS-based technologies.

### 4.1. Class-Diagnostic MS/MS-Based Shotgun Lipidomics

The class-diagnostic MS/MS-based shotgun lipidomics utilizes PIS or NLS or both to monitor one or more class-specific fragments that are typically associated with the head group or the loss of the head group of a lipid class to analyze individual species within the class [37,38]. This approach generally requires at least two internal standards to correct for the effects of differential fragmentation kinetics of individual species for the accurate profiling and quantification. The differential fragmentation kinetics results from the distinct chemical constitution (including acyl chain lengths and unsaturation) of individual species and can lead to species-dependent MS/MS mass spectra after collision-inducted dissociation (CID) [39]. The selection of the two or more internal standards should well represent the chemical structures that span the entire class of interest and a calibration curve is typically determined from the internal standards for the quantification of the species of the entire class.

This quantification method is simple, efficient, and suitable for high throughput lipid analysis. The doubling filtering process of MS/MS enhances the S/N typically by over an order of magnitude. Many laboratories have adopted this approach for profiling and quantifying lipid species. For example, Welti and colleagues have applied this method as an essential tool for plant lipidomics [40]. Hsu and Turk have extensively characterized the fragmentation patterns of various lipid classes and profiled individual species using identified class-specific fragments in multiple classes/subclasses (e.g., subclasses of cerebrosides and choline phospholipids) [41,42]. Hicks and colleagues employed this approach to perform comparative analysis of a variety of phospholipid classes in lipid extracts from distinct tissues of rats and found that each tissue possesses a unique phospholipid signature that can be altered during external perturbations [43]. The specific MS/MS based shotgun lipidomics in combination with stable isotope labeling have been utilized to study the kinetics of lipid turnover, biosynthesis, lipid trafficking and homeostasis and *etc.* because the lipids incorporated with a stable isotope can be easily monitored with PIS of the fragment that contains the labeled tag or NLS of the loss of this fragment [44,45].

The limitations of this approach are also well recognized, including (a) the aliphatic constituents are usually not identified; (b) the presence of isobaric species in a specific MS/MS spectrum cannot be ruled out (*i.e.*, the non-specificity of a class-specific MS/MS due to limited mass accuracy or resolution); (c) the calibration curve based on two or more internal standards cannot fully correct the effects of the differential fragmentation kinetics of various individual species containing differential acyl chain lengths and unsaturations; and (d) the dynamic range of the quantification can be limited if a sensitive diagnostic MS/MS is lacking.

### 4.2. High Mass Accuracy MS-Based Shotgun Lipidomics

The high mass accuracy/mass resolution MS-based shotgun lipidomics generally utilizes hybrid instrumentation such as a Q-TOF or an LTQ Orbitrap mass spectrometer that offers an improved duty cycle [46]. This approach rapidly acquires numerous product ion spectra of individual molecular ions within the mass range of interest or from data-dependent acquisition after direct infusion. From those acquired product ion spectra, multiple precursor ion spectra or neutral loss spectra can be extracted by post acquisition reconstruction. In addition, the high mass accuracy and mass resolution inherent in these instruments allows accurate recording of fragment ion masses that can minimize false-positive identification and facilitate accurate quantification.

In this platform, quantification of individual species can be achieved by comparison of the sum of the intensities of the monitored fragments of a molecular ion to that of the spiked internal standard in the class [47]. The sum of the fragment abundance likely leads to an increased sensitivity of detection and accuracy of quantification. It should be pointed out that ramping collision energies during CID may minimize the effects of differential fragmentation kinetics of discrete species on quantification, and that spiking multiple internal standards for each lipid class may further improve the accuracy of quantification since the platform is essentially dependent on tandem MS. In contrast to the diagnostic MS/MS-based platform, this platform is able to identify and quantify individual lipid species in those lipid classes that do not produce sensitive class-specific fragment ions (e.g., TAG). The linear dynamic range of this approach for quantification is up to four orders of magnitude for most lipid classes [46], which is sufficient for most biological applications.

### 4.3. Multi-Dimensional MS-Based Shotgun Lipidomics

The multi-dimensional MS (MDMS)-based shotgun lipidomics platform maximally exploits the unique chemistries inherent in distinct lipid classes to identify and quantify individual lipid species after direct infusion [4,10,48]. For example, MDMS-based shotgun lipidomics utilizes a multiplexed extraction approach that exploits differential hydrophobicity or differential chemical stability under acidic or basic conditions to separate and/or enrich differential lipid classes by liquid/liquid partitioning or by multiplexed chemistries [10]. MDMS-based shotgun lipidomics also exploits the differential charge properties to achieve selective ionization of differential lipid classes under multiplexed infusion conditions that allow intrasource separation of lipids in different classes or categories [49]. In addition, MDMS-based shotgun lipidomics exploits the uniqueness of individual lipid classes to identify and quantify lipids in specific lipid classes. Examples include quantification of cardiolipins through use of the unique doubly-charged molecular ions resulting from the presence of two phosphate moieties present in cardiolipin resulting in M + 0.5 isotopologue patterns [50]; identification and quantification of phosphoethanolamine-containing lipid species by the specific derivatization of primary amine with fluorenylmethoxylcarbonyl (Fmoc) chloride [51].

MDMS-based shotgun lipidomics utilizes the principle of building block analysis for identification of individual lipid species by employing two powerful MS/MS techniques (*i.e.*, PIS and NLS) in a mass-ramp fashion [10]. Specifically, PIS or NLS of the fragment ion(s) resulting from the head group or the neutral loss of the head group building block identifies the lipid class of interest, and PIS or NLS of fatty acyl building blocks identifies the individual lipid species in the class. The class-specific diagnostic ions are also exploited for lipid quantification. In contrast to the other two shotgun lipidomics platforms, MDMS-based shotgun lipidomics quantifies the identified individual lipid species using a two-step procedure that incorporates not only exogenously added, pre-selected internal standards, but also endogenous lipid species that are quantifiable accurately in a full MS survey scan. Specifically, in the first step, the platform employs a full MS scan acquired after intrasource separation and the pre-selected internal standard of the class of interest for quantification of lipid species that are abundant and not overlapped with lipid species from other classes. Then the platform employs one or more class-specific PIS or NLS spectra and the pre-selected internal standard plus additional standards (that are selected from those endogenous species that have been quantified in the first step) for the quantification of the rest of lipid species in the class that cannot be quantified accurately in the first step due to their low abundance and/or overlapping with species from other classes or impurities from solvents or other sources. In this two-step quantification procedure, both full MS scan and class-specific tandem MS scan(s) as well as both exogenous and endogenous internal standards are used. This leads to a great extension of the accuracy and dynamic range of lipid quantification to the low abundance region due to the use of multiple standards, the elimination of overlapping peaks with class-specific tandem MS scan(s), and the reduced background noise (*i.e.*, increased S/N of low-abundance species). Many lipid classes can be typically achieved [10,52]. An over 5000-fold linear dynamic range has been used to quantify individual species of nearly 30 lipid classes directly from lipid extracts of various biological samples [53].

The second step in MDMS-based shotgun lipidomics is similar to class-specific tandem MS-based shotgun lipidomics for quantification in some aspects. However, the former uses combined exogenous and endogenous standards whereas the latter exclusively uses exogenously added internal standards. The use of endogenous species as standards can generally provide a more comprehensive representation of physical property and chemical composition of individual lipid species over the entire class, while the number of exogenously added internal standards is generally limited in order to eliminate any potential overlapping with endogenous lipid species.

In the case that only two (one exogenous and one endogenous) standards are used in the second step of MDMS-based shotgun lipidomics, this second-step quantification becomes similar to the class-specific tandem MS-based shotgun lipidomics with a linear standard curve which corrects partially the effect of differential acyl chain lengths but not the effect of differential unsaturations of individual species on the quantification. The resultant inaccuracy, however, is relatively small in MDMS-based shotgun lipidomics because its first-step quantification using full MS scan for abundant species can appreciably account for the total content of the class while the class-specific tandem MS-based approach solely relies on the tandem MS spectrum.

The third difference between the second step of MDMS-based approach and the class-specific tandem MS-based approach for quantification is that the MDMS-based approach pre-identifies the species prior to quantification. Therefore, the peaks that are present in the class-specific tandem MS spectrum but without assigned identity are excluded from the second-step quantification, which eliminates the inaccuracy resulting from the possible non-specificity of class-specific tandem MS. If there are more than one class-specific PIS or NLS that are of sufficient sensitivity, they can also be utilized for quantification in the second step to refine the data and serve as an internal check for the accuracy of quantification.

One of the caveats for the second step of MDMS-based platform and the class-specific tandem MS-based platform is that both cannot be applied to a lipid class for which a class-specific and sensitive PIS or NLS is not present (e.g., cardiolipin). Special quantification methods have been developed in MDMS-based shotgun lipidomics for these classes. These methods include derivatizing a moiety of head group to provide a sensitive, class-specific tandem MS (e.g., derivatization of primary amine in head group of ethanolamine-containing classes with Fmoc chloride to allow a facile neutral loss of Fmoc from the tagged species), and exploiting the uniqueness of individual lipid classes (e.g., M + 0.5 isotopologue patterns for doubly-charged cardiolipin species) for quantification.

The other caveat for the second step of MDMS-based shotgun lipidomics is that the species determined in the second step of quantification using endogenous standards quantified in the first step may have a propagated and therefore larger experimental error than the species determined in the first step using exogenously added standard(s). To minimize the error in the second step, it is critical to reduce any potential experimental error in the first step. For example, it is important to use exclusively the species that have large S/N and can be quantified accurately from the first step as endogenous standards for the second step to reduce propagation of errors. Additionally, the propagated experimental error in the second step affects the accuracy of quantification of total amount only moderately because the species quantified in the second step account for a relatively small portion of the total in comparison to those abundant species quantified in the first step.

To validate the quantitative accuracy of the two-step procedure of MDMS-based shotgun lipidomics, we have recently performed a series of experiments by spiking exogenous lipid species before or after extraction to determine the linear dynamic ranges and the matrix effects [10]. In the first set of experiments, a mouse myocardial lipid extract was prepared without addition of any internal standards, and then diluted to a concentration of <100 pmol of total lipids/μL. To the diluted extract solution, different amounts of di14:1 phosphatidylcholine (PC) (commonly used as an internal standard for PC class in the platform) were spiked to reach final concentrations from 0.16 to 16 pmol/μL, spanning a 100-fold range. Considering that the content of numerous endogenous PC species in the myocardial lipid extract spans over 100-fold, this set of experiments tests an overall dynamic range of 10,000 for quantification. The content of di14:1 PC was then separately determined by a full MS scan and two class-specific tandem MS scans (NLS 183.2 and NLS 189.2) with ratiometric comparisons with the base peak at *m/z* 812.6 (*i.e.*, lithiated 16:0-22:6 PC, whose content was pre-determined in a separate lipid extract with the internal standard added prior to extraction). Plotting the spiked content *vs*. the determined content of di14:1 PC from either the full MS spectrum or the tandem MS spectra demonstrated great linear correlations (γ^2^ > 0.997) [10]. In the second set of experiments, a fixed amount of di14:1 PC (15 nmol/mg protein) was used as internal standard and a varied amount of 16:0–18:2 PC (an endogenous species present in mouse myocardial lipid extracts) was added in a factor of its endogenous content (which was pre-determined) from 1- to 100-fold. Both species were added prior to extraction. The content of 16:0–18:2 PC was then separately determined by a full MS scan and two class-specific tandem MS scans (NLS 183.2 and NLS 189.2) with ratiometric comparisons with the internal standard di14:1 PC. Plotting the added content *vs.* the determined content of 16:0–18:2 PC from either the full MS spectrum or the tandem MS spectra also demonstrated great linear correlations (γ ^2^ > 0.998) [10].

Overall, these experimental data validate that the linear dynamic range of quantification is present in either type of scan (survey or tandem) and the matrix effects on quantitation is minimal. Specifically, the linear relationship identified through both full MS and tandem MS are consistent as demonstrated with the small difference in the slope of the regression equations established from both types of scans. Accordingly, these results also validate the accuracy of the two-step quantification procedure utilizing the combination of both full MS scan and class-specific tandem MS scans.

## 5. Concerns Associated with Accurate Quantification

### 5.1. Selection of Internal Standards and Normalization

For an external standard approach, the selected external standard could be the analyte of interest itself because the standard and the analyte are analyzed separately under “identical” conditions. For an internal standard approach where the standard and the analyte are analyzed at the same time, ideal quantification of the analyte can be achieved accurately only if an internal standard chemically identical to the analyte (*i.e.*, its stable isotope-labeled compound) is employed to meet the requirement of identical response factors for standard and analyte in Equation 3. It is obviously impractical to use thousands of stable isotope-labeled internal standards for quantitative analyses of the lipid complex in a cellular lipidome. The finding that the response factors of lipid species by ESI-MS depend predominantly on the electrical properties of the polar head groups in the low concentration region establishes the foundation for employing one species in a lipid class as internal standard to quantify individual lipid species in the class within a reasonable accuracy (approximately 5%) under appropriate conditions (e.g., low concentration region for MDMS-based shotgun lipidomics). The absence of the overlapping of internal standard with the endogenous species of the lipid extract has to be pre-determined using a lipid extract without addition of the internal standard. This is to ensure that the endogenous species overlapping with the internal standard at the spectral resolution is less than 1% of the most abundant species in the class of interest.

For quantification of lipid species in a biological sample, prior to extraction, appropriate amounts of suitable internal standards are added based on a parameter that can be determined accurately and is least varied from sample to sample so that comparison of lipid content between samples can be made. The lipid content of the sample quantified by ratiometric comparison with the internal standards can then be reported after normalization to the parameter. The protein, DNA or RNA content in tissue or cell samples, the tissue wet or dry weight, the cell number, the phosphorus content in the lipid extract, and the volumes of the body fluids are some of the parameters used most often by investigators. Each parameter has benefits and disadvantages. For example, determination of phosphorus content may carry a large experimental error and may be variable under different physiological and pathological conditions. Tissue samples may carry different amounts of water in preparation while it is time-consuming to obtain dry tissue weight. The volume of biofluid may vary with the fluid intake prior to sampling. The cell number counting may become difficult with the presence of aggregated cells. Accordingly, protein or DNA or RNA content as a normalization parameter is highly recommended. Note that although the levels of many proteins change from one state to another, the amounts of the structural proteins that account for most of the protein content of a biological sample do not change significantly.

### 5.2. Aggregation of Lipid Species and Dynamic Range of Quantification

Lipids readily form aggregates (e.g., dimers, oligomers, or micelles) as the lipid concentration increases or the solvent of a lipid solution becomes more polar due to the unique high hydrophobicity of lipid species. The higher the hydrophobicity of a lipid species (e.g., longer acyl chain or less unsaturation), the lower the concentration at which the lipids aggregate. Lipids in aggregated forms cannot be ionized efficiently. Accordingly, lipid species containing short and/or polyunsaturated acyl chains might show higher apparent response factors than those in the same class containing long and/or saturated acyl chains at a concentration that lipid aggregates form. Therefore, lipid aggregation could substantially affect ionization efficiency in a species-dependent fashion. Subsequently, ionization of individual lipid species in a polar lipid class becomes not only charged head group-dependent but also species-dependent, which violates Equation 3. It is, therefore, critical to keep the total lipid concentration lower than the concentration that favors aggregate formation. The maximal lipid concentrations at which lipid aggregation is negligible depend on the solvent system of the lipid solution. The recommended upper limit of total lipid concentration for direct infusion-based approaches is approximately 100 pmol/μL in a 2:1 (v/v), 50 pmol/μL in a 1:1 (v/v), and 10 pmol/μL in a 1:2 (v/v) chloroform-methanol solvent system. However, when an extract contains a large amount of non-polar lipids such as TAG and cholesterol and its esters, this upper lipid concentration limit should be substantially reduced, or alternatively, the upper limit remains for the polar lipid quantification after a pre-fractionation with hexane or other non-polar solvent to remove most of the non-polar lipids from polar lipids. The estimate of the total lipid concentration of a lipid extract is based on pre-knowledge (e.g., approximately 300–500 nmol total lipids/mg of protein for organs such as heart, skeletal muscle, liver, kidney and for some cultured cell types; 1,000–2,000 nmol total lipids/mg of protein for brain samples) or trial experiments when working on an unknown sample with no pre-knowledge.

The effects of lipid aggregation on quantification by direct infusion-based approaches have been appreciated by many investigators. In contrast, the effects of lipid aggregation on quantification by LC-MS-based approaches have been under-estimated. For example, a species eluted from a column is substantially concentrated at its peak time where formation of aggregates (*i.e.*, homo-aggregates from same species) potentially exists. Moreover, the mobile phase used in a reversed-phase HPLC column typically contains polar solvents (e.g., water, acetonitrile, high percentage of methanol, or salts) that favor lipid aggregation in a relatively low concentration. These factors potentially affect the response factors of the lipid species eluted at different times and consequently their quantification especially if only one standard is used.

Dynamic range is always one of the major concerns in quantitative analysis. The detectors used in mass spectrometers generally possess a very wide dynamic range and therefore do not limit the dynamic range for quantitative analysis of lipids. The upper limit of dynamic range, indeed, is the concentration at which the lipids start to form aggregates while the lower limit of dynamic range is the lowest concentration that a method is capable of quantifying individual species (which is generally higher than the limit of detection). This concentration depends on the sensitivity of the instrument, the sensitivity of the method, the effects of matrices and others. For example, LC-MS/MS enhances the S/N through increases of duty cycle and selectivity and typically possesses an extended dynamic range in comparison to LC-MS.

There are at least two different measures of dynamic range. One is the linear range of concentration of the analyte of interest. This measure of dynamic range defines the linear relationship between absolute ion counts and the concentration of a species. As aforementioned, the absolute ion counts of an analyte may be variable and not useful in quantitative analysis of lipids. An alternative way to measure the concentration dynamic range for lipid analysis is to plot the peak intensity ratio of the species of interest and an internal standard in a solution *vs*. the concentration of the solution which spans a wide range of concentration through different folds of dilution. A horizontal line is expected within the linear dynamic range of concentration [18,54]. Another measure of dynamic range is the linear range of the ratio of the species of interest to an internal standard. This can be measured by plotting the peak intensity (or area) ratio in a mass spectrum from direct infusion-based analysis or the extracted peak area ratio from LC-MS-based analysis against the concentration ratio of the species to the standard [18,54]. No more than an approximate 100-fold ratio dynamic range (*i.e.*, from ratio of 0.1 to 10) is normally obtained due to the presence of baseline drift and background noise in full MS spectra, which can dramatically reduce the S/N of low abundance species. Due to the reduced baseline drift and background noise resulting from the double filtering of tandem MS, the use of tandem MS can extend the dynamic range, for example, to 1,000-fold or more depending on the sensitivity of the tandem mass spectra, or even more if a two-step procedure or multiple standards at different ratios are used [10,46,52]. Although the baseline drift and background noise of mass spectra cannot be viewed directly in the SIE chromatogram in LC-MS-based analysis, their presence and effects on quantification of individual species, particularly of low abundance ones, should not be overlooked. For both shotgun lipidomics and LC-MS-based approaches, it is advisable to examine the dynamic range in the presence of sample matrices instead of using a pure standard and under optimized conditions similar to sample analysis to account for the matrix effects (e.g., ion suppression) that become more severe in analysis of minor species (or classes) in the presence of abundant species (or classes).

### 5.3. Correction of ^13^C Isotopologue Effects

Each lipid class in a cellular lipidome is comprised of a variety of lipid species that contain an identical head group but various acyl chains of differential chain length and unsaturation. If an equal molar mixture of the lipid species having differential acyl chains were analyzed by MS, a non-equal monoisotopic peak intensity of the species would be observed with the lower monoisotopic peak intensities observed for the longer acyl chain-containing species due to the differential distribution of isotopologues in those species. Accordingly, the differential isotopologue distribution can affect the lipid quantification by ratiometric comparison with an internal standard if left uncorrected. In general, the isotopologue distribution of each species of a class mainly depends on the number of total carbon atoms in the species because the number of carbon atoms is the most among the atoms present in a species except hydrogen atoms while the effects of differential distribution of H, O, N, P, or other atom-related isotopologues on quantification of lipids are minimal due to either their very low natural abundance or very small atom numbers in a species relative to carbon atom number or both. Therefore, the correction of ^13^C isotopologue effects is mainly discussed below. The isotopologue effects of other atoms can be included if necessary with more comprehensive algorithms [55,56]. However, when the atoms such as Cl or S whose isotopologues have big natural abundance are present in a species, the effects of their isotopologue distribution on quantification are not negligible and have to be taken into account carefully.

There are two types of ^13^C isotope corrections. The first one is to sum the intensities of all the isotopologues for each species including the internal standard. Quantification by ratiometric comparison with internal standard is based on the ratio of the sum of the isotopologue intensities of a species to that of the internal standard. The mono-isotopic peak is the most intense peak in the isotopologue cluster of a lipid species for almost all lipids and its intensity can therefore be determined more accurately compared to the intensities of other isotopic peaks of the species. Meanwhile, the intensity of each isotopologue of a species can be easily deduced from the determined mono-isotopic peak intensity. Therefore, the first correction factor can be derived as follows. The total ion intensity (I_total(n)_) of an isotopologue cluster of a lipid species is (Equation 4):

(4) $${\text{I}}_{\text{total}(\text{n})}={\text{I}}_{\text{n}}(1+0.0109\text{n}+0.0109^{2}\text{n}(\text{n}-1)/2+\ldots )$$

where I_n_ is the mono-isotopic peak intensity of the species containing n carbon atoms and 0.0109 is the abundance of ^13^C in nature when the abundance of ^12^C is defined as 1. For quantification of this species with an internal standard containing s carbon atoms, we have when conditions of Equation 3 are satisfied:

(5) $$\begin{array}{c} {\text{C}}_{\text{n}}={\text{I}}_{\text{total}(\text{n})}/{\text{I}}_{\text{total}(\text{s})}*{\text{C}}_{\text{s}}\\ =(1+0.0109\text{n}+0.0109^{2}\text{n}(\text{n}-1)/2+\ldots ){\text{I}}_{\text{n}}/(1+0.0109\text{s}+0.0109^{2}\text{s}(\text{s}-1)/2+\ldots ){\text{I}}_{\text{s}}*{\text{C}}_{\text{s}}\\ ={\text{Z}}_{1}*({\text{I}}_{\text{n}}/{\text{I}}_{\text{s}})*{\text{C}}_{\text{s}} \end{array}$$

Where

(6) $$Z_{1}=(1+0.0109\text{n}+0.0109^{2}\text{n}(\text{n}-1)/2+\ldots )/(1+0.0109\text{s}+0.0109^{2}\text{s}(\text{s}-1)/2+\ldots )$$

and is called the type I ^13^C isotope correction factor; n and s are the numbers of total carbon atoms in the species of interest and in the selected internal standard, respectively; I_n_ and I_s_ are the mono-isotopic peak intensities of the species and the internal standard, respectively; C_n_ and C_s_ are the concentration of the species of interest and the internal standard, respectively. The dots represent the contribution of other isotopologues which contain more than two ^13^C atoms. These terms can be ignored in most cases without affecting the accuracy of quantification due to their extremely small contributions compared to the contributions from the monoisotopic, the second, and the third isotopic peaks. It should be mentioned that, following this line of reasoning, a method based on comparison of the intensities between cardiolipin M + 1 (*i.e.*, the second) isotopologues, which exploits the uniqueness of doubly-charged cardiolipin ions, has been developed and is very powerful for quantification of individual cardiolipin molecular species [50,57].

The second type of ^13^C isotope correction results from the fact that the monoisotopic peak of the species of interest is isobaric with the second isotopologue of a species that differs from the species of interest with only one more double bond. It is obvious that this type of correction is not needed if the aforementioned isobaric peaks can be resolved with high mass resolution instrumentation. If the overlapping from the isobaric peaks cannot be resolved (e.g., when low to moderate resolution mass spectrometers are used), corrections on the apparent monoisotopic peak intensities I_n_′ and I_s_′ are needed to obtain the actual monoisotopic peak intensities I_n_ and I_s_ for the Equation 5. The correction on I_n_′ is derived as follow as an example and the correction on I_s_′ can be done similarly.

(7) $$\begin{array}{c} {\text{I}}_{\text{n}}={\text{I}}_{\text{n}}\prime -{\text{I}}_{\text{N}}*(0.0109^{2}\text{n}(\text{n}-1)/2)\\ =(1-({\text{I}}_{\text{N}}/{\text{I}}_{\text{n}}\prime )(0.0109^{2}\text{n}(\text{n}-1)/2))*{\text{I}}_{\text{n}}\prime \\ =Z_{2}*{\text{I}}_{\text{n}}\prime \end{array}$$

Where

(8) $$Z_{2}=1-({\text{I}}_{\text{N}}/{{\text{I}}_{\text{n}}}^{\prime })(0.0109^{2}\text{n}(\text{n}-1)/2)$$

and is called the type II ^13^C isotope correction factor; n is the number of total carbon atoms in the species of interest and I_n_′ is the apparent mono-isotopic peak intensity of the species; I_N_ is the mono-isotopic peak intensity of the species that differs from the species of interest with only one more double bond; and I_n_ is the corrected monoisotopic peak intensity of the species of interest. This correction factor can be negligible if I_N_ ≪ I_n_′.

It should be specifically pointed out that when a tandem MS spectrum is used for quantification using Equation 5 in which I_n_ and I_s_ are obtained after isotope correction using Equation 7 and a similar one, respectively, both types of correction factors (*i.e.*, *Z*_1_ and *Z*_2_) may need to be modified because the fragment monitored in tandem MS (*i.e.*, the fragment ion in PIS or the neutral fragment in NLS) is the monoisotopic one and therefore contains ^12^C atoms only. Accordingly, the number of total carbon atoms in Equations 6 and 8 should be deduced by subtraction of the number of the carbon atoms in the monitored fragment that contribute no ^13^C isotopologue effects. It should also be pointed out that if a calibration curve using two or more internal standards covering a wide mass range is used (e.g., in the class-specific tandem MS-based shotgun lipidomics), the first type of correction factor (*Z*_1_) can be largely covered by the calibration curve but the second type of correction factor (*Z*_2_) should still be considered. In LC-MS based approaches, if the chromatographic separation can totally resolve individual lipid species in a class and a calibration curve is established for each individual species, both correction factors are not needed. Otherwise, these corrections or other alternative de-isotoping should always be taken into account.

## 6. Conclusions

ESI-MS analysis of lipid is the most prominent approach and has enjoyed the most success in lipidomics. With great efforts of the researchers in the field, a complete quantitative analysis of lipid classes, subclasses, and individual molecular species by using ESI-MS with or without chromatographic separation is possible. However, it is very important to understand the principles of quantitation by MS, learn the limitations of each platform for lipid analysis, and keep the general concerns in mind so that an accurate result can be obtained and a meaningful conclusion can be drawn. It is our sincere hope that with our precautions, we can successfully meet one of the major challenges (*i.e.*, accurate quantification of individual lipid species by MS) in lipidomics.
